# A Combined Proteomic and Transcriptomic Analysis on Sulfur Metabolism Pathways of *Arabidopsis thaliana* under Simulated Acid Rain

**DOI:** 10.1371/journal.pone.0090120

**Published:** 2014-03-03

**Authors:** Tingwu Liu, Juan A. Chen, Wenhua Wang, Martin Simon, Feihua Wu, Wenjun Hu, Juan B. Chen, Hailei Zheng

**Affiliations:** 1 Key Laboratory of the Ministry of Education for Coastal and Wetland Ecosystems, College of the Environment and Ecology, Xiamen University, Xiamen, Fujian, P. R. China; 2 Department of Biology, Huaiyin Normal University, Huaian, Jiangsu, P. R. China; 3 Department of Biology, Duke University, Durham, North Carolina, United States of America; 4 State Key Laboratory of Marine Environmental Science, Xiamen University, Xiamen, Fujian, P. R. China; National Taiwan University, Taiwan

## Abstract

With rapid economic development, most regions in southern China have suffered acid rain (AR) pollution. In our study, we analyzed the changes in sulfur metabolism in *Arabidopsis* under simulated AR stress which provide one of the first case studies, in which the systematic responses in sulfur metabolism were characterized by high-throughput methods at different levels including proteomic, genomic and physiological approaches. Generally, we found that all of the processes related to sulfur metabolism responded to AR stress, including sulfur uptake, activation and also synthesis of sulfur-containing amino acid and other secondary metabolites. Finally, we provided a catalogue of the detected sulfur metabolic changes and reconstructed the coordinating network of their mutual influences. This study can help us to understand the mechanisms of plants to adapt to AR stress.

## Introduction

Acid rain (AR), as a worldwide environmental issue, has been a serious global problem for several decades, especially in southern China [1]. As for plants, it has caused a series of damages, such as necrosis, thin crown, premature abscission, branch dieback, and has been treated as a new abiotic stress factor [2]–[4]. Acid rain is formed from SO_2_ and nitrous oxides (NOx) emitted to the atmosphere, largely due to fossil-fuel combustion [5]. Different from other regions in the world, AR in China contains a lot of sulfate [1] due to the aggravated combustion of ubiquitous sulfur-containing coal [6]. As a result of significant emissions and subsequent deposition of sulfur (S), widespread AR is observed in southern and southwestern China [1]. However, studies are rarely focused on the plant's response in S metabolism to AR, and molecular details of this process are poorly understood [7].

S is an essential mineral element that is required in large amount in plants, animals, and microorganisms [8]. It is uptaken as sulfate and is then assimilated into organic compounds. S is found in two amino acids including cysteine (Cys) and methionine (Met), in oligopeptides including glutathione (GSH) and phytochelatins, in some vitamins and cofactors including biotin, molybdenum cofactor, thiamine and coenzyme A, in phytosulfokin hormones and in a variety of secondary products, all of which are essential in plant nutrition [9]. Finally, S is integrated into some S-containing proteins. S also plays a critical role in catalytic and electrochemical functions in these biomolecules. Disulfide bonds between polypeptides, mediated by Cys, are of great importance in protein assembly and structure [10]. The regulation of sulfate uptake and assimilation has been dissected in great detail [11]–[13], and dynamic adaptations of the integrative gene-metabolite network in response to S deficiency have been deciphered [12], [14], [15].

Proteomic, transcriptomic, and metabolomic approaches can provide the comprehensive profiles of large numbers of gene expression products [16]. The use of these approaches to obtain comprehensive data sets increased rapidly in recent years, especially with respect to the mechanisms underlying plant growth and plant responses to stress [14], [17]. The new high-throughput tools have provided the potential to systematically analyze biological systems and monitor their responses. By conceiving the network architecture and thus the interrelation and regulation of its components, it can be envisioned that it will be possible to comprehend the whole system.

In the present study, we explored whole-cellular processes of S metabolism at the levels of transcriptome and proteome in *Arabidopsis* under AR stress by applying a DNA array and a combination of proteomic and transcrpimic analysis. We depicted a whole picture for the changes of plant S metabolism under AR by combining an amount of multidimensional data. These data can provide novel indications as to reveal the response of the processes related to S metabolism to AR at the levels of the transcriptome and proteome.

## Materials and Methods

### Plant Materials and Growth Conditions

Seeds of *Arabidopsis thaliana*, ecotype Columbia-0 (Col-0) were planted in the mixed matrix with vermiculite and cover soil (2∶1) after vernalization. Then, plants were grown in controlled growth chamber with a light/dark regime of 16/8 hr, temperature of 23/20°C and a light intensity of 150 µmol m^−2^ s^−1^ photosynthetically active radiation (PAR). After 3 weeks, the seedlings were sprayed by simulated acid rain (AR, pH 3.0) at 5 ml per seedling, meanwhile, the seedlings were sprayed with control solution (CK, pH 5.6) which had the same ion composition as AR. The AR solution was prepared from H_2_SO_4_ and HNO_3_ in the ratio of 5 to 1 by chemical equivalents, which represents the average ion composition of rainfall in South China [18]. The final concentrations of H_2_SO_4_ and HNO_3_ in the spray solution were 0.45 and 0.09 mM, respectively. The leaves were collected after AR treatment for 3 days and they were immediately frozen in liquid nitrogen (N_2_) and stored at 70°C for subsequent protein/RNA extraction and enzyme for protein and RNA extraction assays. The phenotype of the treated and control groups were shown in Figure S1 after AR treatment. Each experiment was repeated at least three times.

### Microarray Analysis

For Affymetrix GeneChip analysis, the materials were treated the same as described above. 20 mg of total RNA from leaves of *Arabidopsis* with or without AR treatment was extracted using the RNeasy plant mini kit (Qiagen), and the product was used to make biotin-labeled cRNA targets. The Affymetrix *Arabidopsis* ATH1 genome array GeneChip, which contains >22,500 probe sets representing-24,000 genes, was used. Hybridization, washing, and staining were performed according to the manufacturer's instructions. Image processing was performed using Affymetrix GeneChip Operating System (GCOS). Normalization and expression estimate computation were calculated from the. CEL output files from the Affymetrix GCOS 1.1 software using RMA implemented in R language using standard settings. Statistical testing for differential expression was performed with logic-t analysis. All microarray expression data are available at the Gene Expression Omnibus under the series entry GSE52487. Functional categories were assigned to genes using the AGI number to search the MIPS database (http://mips.gsf.de/cgi-bin/proj/thal/) and the *Arabidopsis* Information Resource website, TAIR (http://www.arabidopsis.org/).

### Total Protein Extraction and Two-dimensional Electrophoresis

Proteins were extracted under denaturing conditions, according to the phenol procedure [19]. Briefly, one gram of frozen lyophilized tissue powder was re-suspended in 3 mL ice-cold extraction buffer (100 mM PBS, pH 7.5) containing 100 mM EDTA, 1% PVPP w/v, 1% Triton X-100 v/v, 2% b-mercaptoethanol v/v. After centrifugation at 4°C, 15,000 *g* for 15 min, the upper phase was transferred to a new centrifuge tube. Two volumes of Tris-saturated phenol (pH 8.0) were added and then the mixture was further vortexed for 10 min. Proteins were precipitated by adding five volumes of ammonium sulfate saturated-methanol, and incubating at −20°C for at least 4 h. After centrifugation as described above, the protein pellet was re-suspended and rinsed with ice-cold methanol followed by ice-cold acetone twice, and spun down at 15,000 *g* and 4°C for 5 min after each washing. Finally, the washed pellets were air-dried and recovered with lysis buffer containing 7 M urea, 2 M thiourea, 2% CHAPS, 13 mM DTT and 1% IPG buffer. The sample containing 800 µg of total proteins was subsequently loaded onto an IPG strip holder with length 17 cm, pH 4–7 linear gradient IPG strips (GE Healthcare, Sweden), and rehydrated for 24 h at room temperature. Strips were covered with mineral oil to prevent evaporation. Then IEF was performed as the following: 300 V for 1 h, 600 V for 1 h, 1000 V for 1 h, a gradient to 8000 V for 2 h, and kept at 8000 V for 64,000 V·h. After focusing, the strips were equilibrated with equilibration solution (50 mM Tris, pH 8.8, 6 M urea, 30% glycerol, 2% SDS) containing 1% DTT, and subsequently 4% iodoacetamide for 15 min for each equilibration solution. The separation of proteins in the second dimension was performed with SDS polyacrylamide gels (12%) on an Ettan DALT System (GE Healthcare, Sweden) and sealed in with 0.5% agarose, and run at 10 mA for electrophoresis. Each separation was repeated 3 times to ensure the protein pattern reproducibility.

### Protein Staining and Image Analysis

The SDS-PAGE gels were stained by the CBB R250. 2-DE gels were scanned at 600 dots per inch (dpi) resolution with a scanner (Uniscan M3600). 2-D gel analysis was performed by PDQuest software (Bio-Rad). For each gel, a set of three images was generated, corresponding to the original 2-D scan, the filtered image, and the Gaussian image. The Gaussian image containing the three-dimensional Gaussian spots was used for the quantification analysis. The intensity of each protein spot was normalized relative to the total abundance of all valid spots. After normalization and background subtraction, a matchset was created by comparing the control gels. All spots were then submitted to further analysis to test whether or not their expression levels were affected by AR treatment and those that increased or decreased significantly more than 2-fold change were then identified by MALDI TOF/MS. The apparent *M*r of each protein in gel was determined with protein markers.

### Protein Identification

Excised gel spots were washed several times with destaining solutions (25 mM NH_4_HCO_3_ for 15 min and then with 50% v/v ACN containing 25 mM NH_4_HCO_3_ for 15 min). Gel pieces were dehydrated with 100% ACN and dryed, then incubated with a reducing solution (25 mM NH_4_HCO_3_ containing 10 mM DTT) for 1 h at 37°C, and subsequently with an alkylating solution (25 mM NH_4_HCO_3_ containing 55 mM iodoacetamide) for 30 min at 37°C. After reduction and alkylation, gels were washed several times with the destaining solutions and finally with pure water for 15 min, before dehydration with 100% ACN. Depending on protein amount, 2–3 µL of 0.1 mg µL^−1^ modified trypsin (Promega, sequencing grade) in 25 mM NH_4_HCO_3_ was added to the dehydrated gel spots. After 30 min incubation, 7 µL of 25 mM NH_4_HCO_3_ were added to submerge the gel spots at 37°C overnight.

After digestion, the protein peptides were collected and vacuum-dried. 0.5 µL peptide mixture was mixed with 0.5 µL matrix solution (HCCA at half saturation in 60% ACN/0.1% TFA v/v). A total of 1 µL of reconstituted in-gel digest sample was spotted initially on Anchorchip target plate. The dried sample on the target plate was washed twice with 1 µL of 0.1% TFA, left for 30 s before solvent removal. MALDI TOF MS analysis (ReFlexTMIII, Bruker) was used to acquire the peptide mass fingerprint (PMF). The spectra were analyzed with the flexAnalysis software (Bruker-Daltonics). All spectra were smoothed, and internally calibrated with trypsin autolysis peaks. Then, the measured tryptic peptide masses were transferred through MS BioTool program (Bruker-Daltonics) as inputs to search against the taxonomy of *Arabidopsis thaliana* (thale cress) in NCBI (NCBInr) database. The PMF searched parameters were 100 ppm tolerance as the maximum mass error, MH^+^ monoisotopic mass values, allowance of oxidation (M) modifications, allowed for one missed cleavage, and fixed modification of cysteine by carboxymethyl (Carbamidomethylation, C). The match was considered in terms of a higher Mascot score, the putative functions, and differential expression patterns on 2-DE gels. Good matches were classified as those having a Mascot score higher than 60 (threshold). The identification was considered only with a higher MASCOT score, maximum peptide coverage and additional experimental confirmation of the protein spots on the 2-DE gels. The identified proteins were searched within the UniProt and TAIR database to find out if their function was known, then they were further classified using Functional Catalogue software (http://mips.gsf.de/projects/funcat).

### Real-time Quantitative PCR

Verification of differential gene expression was performed by real-time quantitative PCR (qRT-PCR) in the Rotor-GeneTM 6000 real-time analyzer (Corbett Research, Mortlake, Australia) using the FastStart Universal SYBR Green Master (ROX, Roche Ltd., Mannheim, Germany) according to the manufacturer's instructions. Reaction conditions (10 µL volumes) were optimized by changing the primer concentration and annealing temperature to minimize primer-dimer formation and to increase PCR efficiency. The following PCR profile was used: 95°C for 5 min, 40 cycles of 95°C for 30 s, the appropriate annealing temperature for 30 s and 72°C for 30 s, a melting curve was then performed to verify the specificity of the amplification. Each run included standard dilutions and negative reaction controls. Successive dilutions of one sample were used as a standard curve. All the results presented were standardized using the housekeeping gene *Actin2.* The results of the mRNA expression level of genes were expressed as the normalized ratio using the _ΔΔ_Ct method according to Livak and Schmittgen [20]. Ct values of each target gene were calculated by Rotor-Gene 6000 Application Software, and the _Δ_Ct value of the *Actin2* rRNA gene was treated as an arbitrary constant for analyzing the _ΔΔ_Ct value of samples. Three independent pools for each target gene were averaged, and the standard error of the mean value was recorded. The primer sequences used for the gene amplification are described in Table S1.

### Physiological Index

#### Glutathione (GSH) Content

Glutathione (GSH) Content was estimated fluorimetrically according to Karni et al [21]. Half a gram plant material was frozen in liquid nitrogen and ground in 0.5 mL of 25% H_3_PO_3_ and 1.5 mL of 0.1 M sodium phosphate-EDTA buffer (pH 8.0). The homogenate was centrifuged at 10,000 *g* for 20 min to obtain supernatant for the estimation of GSH. The supernatant was diluted four times with phosphate-EDTA buffer (pH 8.0). The assay mixture for GSH estimation contained 100 mL of the diluted supernatant, 0.9 mL of phosphate-EDTA buffer and 100 mL of *O*-phthalaldehyde solution (1 mg : 1 mL). After thorough mixing and incubation at room temperature for 15 min, the solution was transferred to a quartz cuvette and the fluorescence at 420 nm was measured after excitation at 350 nm.

#### Ser Acetyltransferase Activity

Ser acetyltransferase (SAT) activity was measured according to the method described by Youssefian et al [22]. The incubation mixture with final volume of 240 µL contained 12 µM KPO_3_, 16 µM Ser, 30 µg BSA, 0.5 µM acetyl CoA, 1 µM Na_2_S, and an appropriate amount of extracts. The reaction was started by addition of the extracts and continued for 20 min at 25°C and was terminated by addition of 400 µL 4 M HCl. The tubes were centrifuged at 15,000 *g* for 3 min and to an aliquot of 200 µL supernatant, 200 µL modified ninhydrine reagent was added. The mixture was heated at 100°C for 10 min and cooled rapidly on ice, then 400 µL 98% ethanol was added and the absorbance was determined at 560 nm. The calibration curve was established by adding known amounts of L-Cys to the assay mixture and measuring these without incubation.

#### Amino Acid Content

The samples of plant material (0.5 g) were mixed with 1 ml of extraction solution (60% methanol, 25% chloroform, and 15% water) at 42°C for 10 min. After brief centrifugation, the supernatant was collected and the residue was extracted with the same mixture solution again, then both supernatants were combined. After adding the chloroform (1 mL) and water (1 mL), the resulting mixture was centrifuged again and the upper water-methanol phase was collected. Then the supernatants were dried in a vacuum desiccator, and then dissolved in 200 µL of water. The concentration of free amino acids was determined using *O*-phthalaldehyde reagent, followed by measuring the 335/447 nm fluorescence. Amino acid analyses were performed by the ion-exchange chromatography technique with a Hitachi model L-8800 amino acid analyzer (Hitachi Co. Ltd., Tokyo, Japan) with a column packed with Hitachi custom ion-exchange resin.

### Statistical Analysis

Each experiment was repeated at least three times. Values in figures and tables were expressed as means ± SE. The statistical significance of the data was analyzed using univariate analysis of variance (*p*<0.05) (one-way ANOVA; SPSS for Windows, version 11.0).

## Results and Discussion

### Integrative Proteomic and Transcriptomic Analysis on S Metabolism

**Figure 1:**
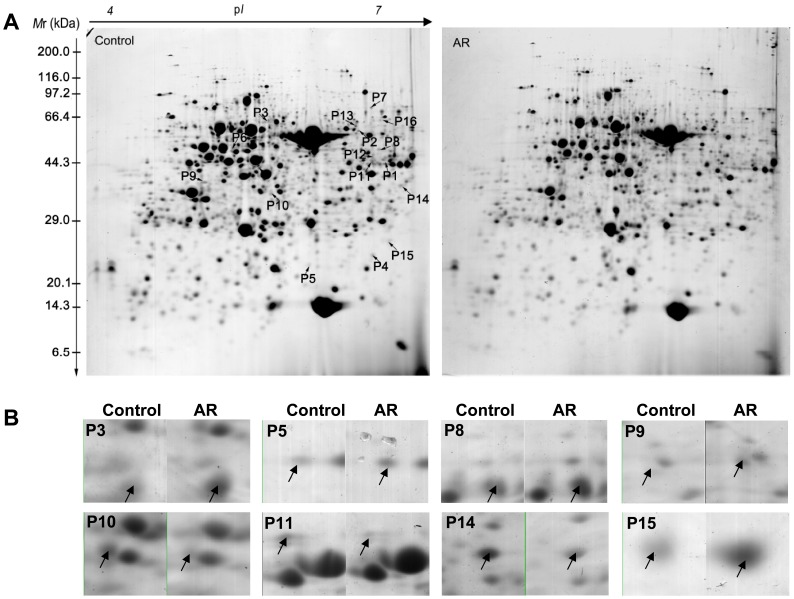
Protein expressions of *Arabidopsis thaliana* leaves after simulated acid rain (AR) treatment for 3 days (A). Molecular weight (*M*r) in kilodaltons and p*I* of proteins are indicated on the left and top of the representative gel, respectively. Sixteen spots related to sulfur metabolism with at least a 2-fold change under AR stress are indicated. Close-up view of some differentially expressed protein spots (B).

**Figure 2:**
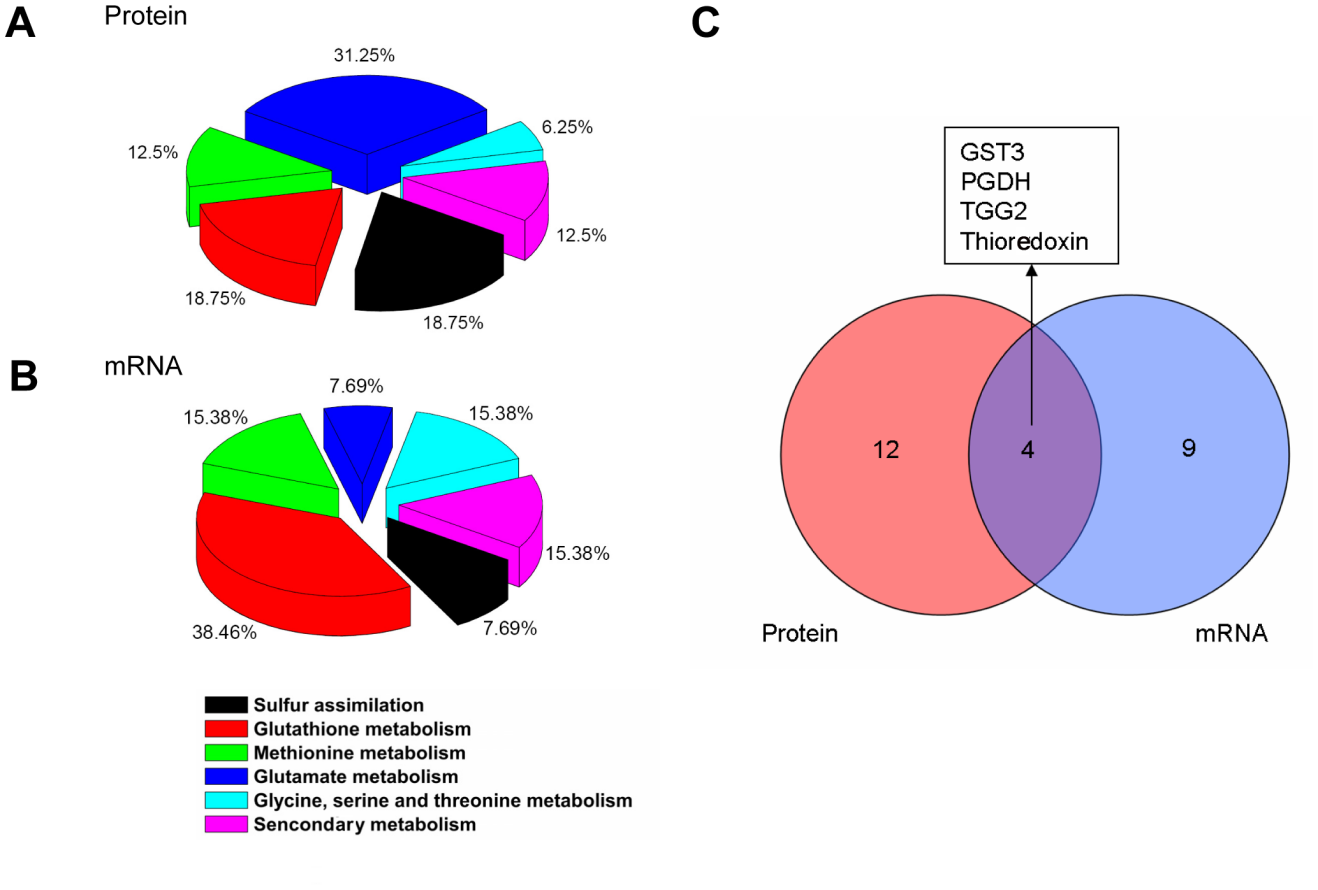
Functional classification of the significant differential expression proteins (A) and genes (B) after simulated acid rain (AR) treatments in *Arabidopsis thaliana*. Venn diagram shows the number of overlapped genes or proteins between gene and protein expression profiles after AR treatment (C).

In order to investigate the expression changes of proteins related to S metabolism under AR treatment, we analyzed the expression patterns of AR responsive proteins using a proteomic approach. The proteins were separated by 2-DE. On CBB-stained 2-DE gels, over 1500 highly reproducible protein spots in the p*I* range of 4–7 were revealed. 2-DE maps of the leaf proteome are shown in Figure 1A. Close-up views of several protein spots are shown in Figure 1B. Sixteen proteins related to S metabolism were identified and thereafter the functional categories were assigned to proteins using the AGI number to search the MIPS database (Figure 2A). Detailed information including the description of proteins, the MOWSE scores, theoretical p*I* values, molecular weights (*M*r) and peptides matched of those 16 proteins which are related to S assimilation and primary/secondary metabolism are shown in Table 1 and Table S2.

**Table 1:** Identification of protein spots with a significant 2-fold changes in AR compared with control treatment for *Arabidopsis thaliana* leaves.

Spot	Accession number	Protein identity	Theo. M_r_ (kDa)/p*I*	Expt. M_r_ (kDa)/p*I*	SC (%)	Mascot score	Fold change	SP/TP	p-value
	**Sulfur metabolism**								
P1	AT4G14880	O-acetylserine (thiol) lyase (OASA1)	34/5.9	42/7.0	32%	88	−3.6±0.21	5/13	1.1×10^−3^
P2	AT2G43750	(OAS-TL) isoform oasB (OASB)	42/8.2	42/7.0	21%	89	−4.0±0.30	6/15	9.8×10^−6^
P3	AT3G22890	ATP sulfurylase (APS)	51/6.8	51/6.3	23%	91	2.4±0.12	7/13	4.8×10^−16^
	**Glutathione metabolism**								
P4	AT5G41670	6-phosphogluconate dehydrogenase family protein (6-PDG)	53/5.5	54/5.6	19%	71	3.5±0.22	6/16	1.6×10^−4^
P5	AT5G16710	glutathione dependent dehydroascorbate reductase (DHAR3)	29/7.9	28/7.0	41%	68	4.4±0.31	11/31	6.0×10^−13^
P6	AT1G02920	glutathione S-transferase (GST3)	24/6.6	24/6.1	50%	67	2.9±0.16	8/29	1.2×10^−9^
	**Methionine metabolism**								
P7	AT3G17390	S-adenosylmethionine synthetase (MTO3)	43/5.6	43/5.5	46%	95	2.3±0.14	15/32	7.6×10^−9^
P8	AT5G17920	Methionine synthase (MetS)	84/6.5	85/6.1	31%	139	2.3±0.11	17/24	1.5×10^−4^
	**Glutamate metabolism**								
P9	AT1G23310	glutamate-glyoxylate aminotransferase (GGT1)	53/6.9	49/6.6	24%	87	2.1±0.05	21/36	1.2×10^−3^
P10	AT1G66200	glutamine synthase clone R2 (GSR2)	39/4.9	39/5.1	28%	95	−2.4±0.14	10/17	1.5×10^−2^
P11	AT3G17820	glutamate-ammonia ligase (GLD)	39/5.9	39/5.7	47%	92	−3.0±0.18	14/22	6.1×10^−7^
P12	AT5G63570	glutamate-1-semialdehyde 2,1-aminomutase (GSA)	50/6.9	51/6.4	27%	99	−3.9±0.21	11/27	1.5×10^−13^
P13	AT3G17240	lipoamide dehydrogenase (LPD)	54/7.0	54/6.6	32%	86	2.9±0.22	10/23	5.6×10^−3^
	**Glycine, serine and threonine metabolism**								
P14	AT4G34200	phosphoglycerate dehydrogenase (EDA9)	63/6.5	64/6.3	23%	106	−3.3±0.20	15/27	6.5×10^−2^
	**Others**								
P15	AT4G03520	thiol-disulfide exchange intermediate (TRX5)	20/9.6	21/6.9	34%	90	4.0±0.18	5/9	2.4×10^−9^
P16	AT5g25980	thioglucoside glucohydrolase (TGG2)	63/7.5	62/7.1	33%	180	3.6±0.14	14/21	2.1×10^−4^

To further examine the responses of *Arabidopsis* to AR, we applied transcript profiling employing the Affymetrix AH1 chips covering 24,000 genes to analyze the changes in gene expression patterns. In total, 13 genes which dramatically changed their expression were found related to S metabolism (Table 2). A list of the 13 S metabolism related genes significantly regulated at the transcript level, having been re-annotated and classified into functional classes as defined by MIPS database, is provided in Table 2.

**Table 2:** Differentially expressed transcripts induced by AR for *Arabidopsis thaliana* leaves.

Probe ID	NO.	Gene name	Accession number	Description	Fold change	*p*-Value
	**Sulfur metabolism**					
260602_at	G1	SAT	At1g55920	Serine acetyltransferase	2.3±0.03	4.3×10^−4^
	Glutathione metabolism					
266746_s_at	G2	GSTF3	At2g02930	Glutathione S-transferase	2.3±0.06	3.7×10^−3^
264383_at	G3	GPX1	At2g25080	Glutathione peroxidase	2.7±0.02	6.5×10^−5^
254890_at	G4	GPX6	At4g11600	Ggutathione peroxidase	2.5±0.01	6.2×10^−5^
246785_at	G5	GSH2	At5g27380	Glutathione synthetase	2.3±0.04	1.7×10^−3^
262932_at	G6	Micro-GST	At1g65820	Microsomal glutathione S-transferase	2.0±0.05	1.7×10^−3^
	Glutamate metabolism					
249581_at	G7	GSR1	At5g37600	Cytosolic glutamine synthetase	−2.4±0.03	3.2×10^−4^
260309_at	G8	AOAT2	At1g70580	Glutamate-glyoxylate transaminase	−2.6±0.07	1.1×10^−2^
	Methionine metabolism					
246490_at	G9	SAMDC	At5g15950	S-adenosylmethionine decarboxylase	−3.5±0.07	1.3×10^−3^
	Glycine, serine and threonine metabolism					
253162_at	G10	PSAT	At4g35630	Phosphoserine amintransferase	−2.5±0.02	2.4×10^−4^
259403_at	G11	PGDH	At1g17745	Phosphoglycerate dehydrogenase	−2.5±0.01	1.3×10^−4^
	Other metabolism					
260943_at	G12	TRX5	At1g45145	Cytosolic thioredoxin	3.3±0.06	1.9×10^−2^
265058_s_at	G13	TGG2	At1g52040	Thioglucoside glucohydrolase	5.4±0.09	2.1×10^−2^

From our results, the differentially expressed proteins and genes under AR covered each step of S metabolism pathways according to their functional categories, including S uptake, transportation, reduction, assimilation and S-containing amino acids and other derivates synthesis metabolisms (Figure 2A and B). A Venn diagram of regulated cytosolic mRNA versus regulated proteins shows an overlap of 4 genes (Figure 2C), indicating that a large number of genes are solely regulated either at mRNA or protein level. Similar results were also found in earlier studies [14], [17], [23], [24]. Here are some reasons that may clarify the results. Firstly, proteomic studies suffer from inherent technical shortcomings associated with, for example, protein insolubility, fractionation losses, extreme p*I*, etc [17]. Secondly, despite recent improvements, proteomic technique remains poorly suitable to separate highly hydrophobic, basic or low-abundant proteins [23]. Thus, subcellular membrane proteome, and especially their integral protein moieties, remain poorly accessible [25], [26]. On the other hand, mRNA degradation, alternative splicing, and post-transcriptional regulation of gene expression could also lead to the lack of strong correlations with protein expression status [27].

**Figure 3:**
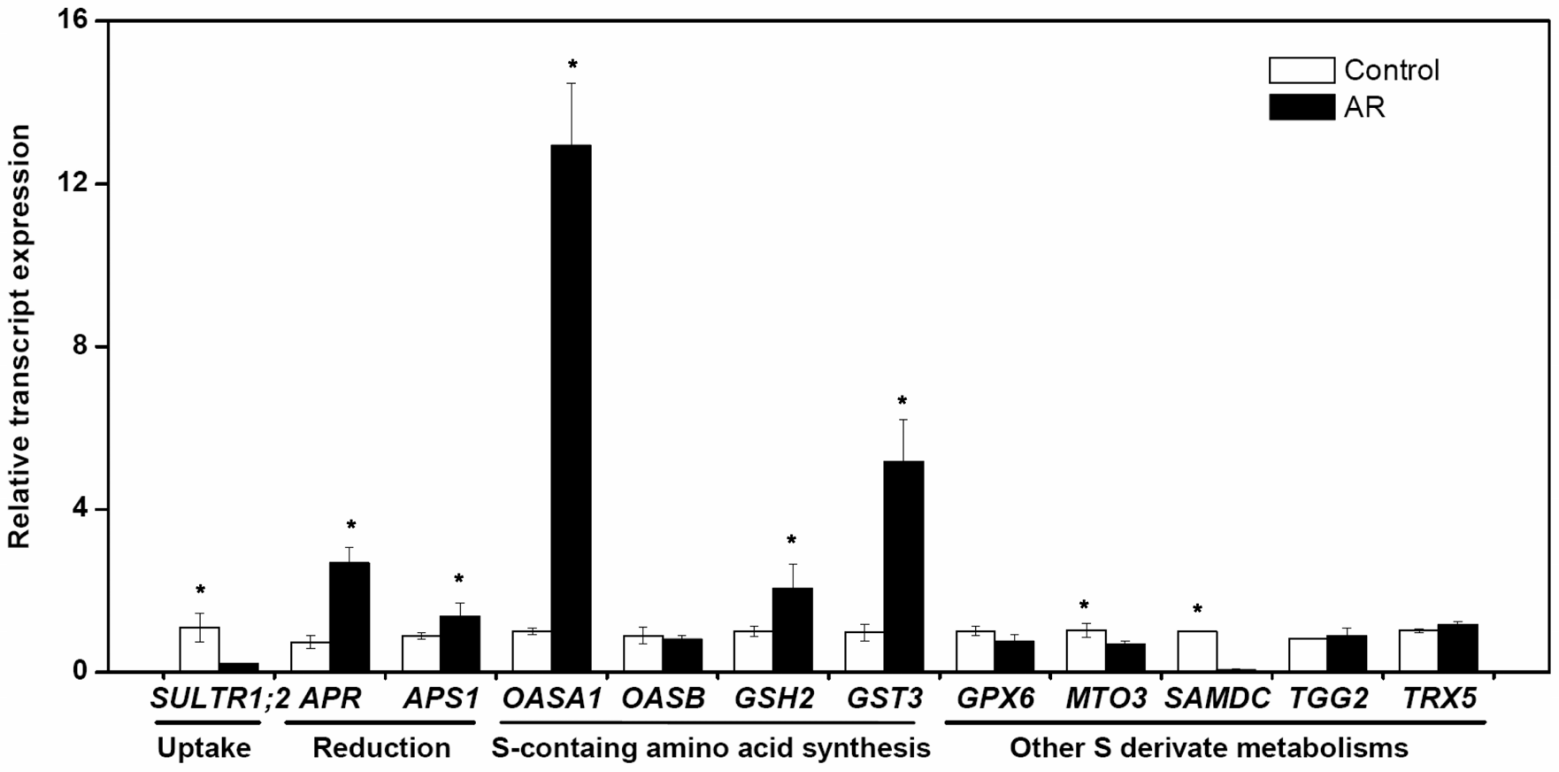
Relative changes in transcript level quantified by qRT-PCR for twelve genes related to sulfur metabolism in *Arabidopsis thaliana* leaves under simulated acid rain (AR). The fold-change values were derived from the average of three replicate measurements. The asterisk indicates significance at *p*<0.05.

In order to further confirm and extend the results obtained from proteomic and transcriptomic analysis, we performed quantitative real-time PCR (qRT-PCR) analysis on 12 genes, all of which are very crucial in S metabolism pathways, including S uptake (Sulfate transporter1;2 gene, *SULTR1;2*), reduction (ATP sulfurylase gene, *APS*; APS reductase gene, *APR*) as well as on the genes related to S-containing amino acids synthesis (*O*-acetylserine(thiol)lyase gene, *OASA*; Cysteine synthase gene, *OASB*; Glutathione synthetase gene, *GSH2*) and other S derivates synthesis metabolisms (Glutathione S-transferase gene, *GST3*; Glutathione peroxidase gene, *GPX6*; Cytosolic thioredoxin gene, *TRX5*; Myrosinase gene, *TGG2*; S-adenosylmethionine synthetase gene, *MTO3*; S-adenosylmethionine decarboxylase gene, *SAMDC*). qRT-PCR analysis showed that transcript expression level of genes related to primary sulfur assimilation, such as *APR, APS1, OASA1 GSH2* and *GST3*, were up-regulated (Figure 3). However, the synthesis genes of some S-containing amino acids and derivatives (*MTO3* and *SAMDC*) were down-regulated (Figure 3). The results were highly correlated with those of the array data, thus confirming the results from proteomic and transcriptomic studies. However, the change level of differential expression of a single gene was a little different with microarray as described previously [14]

### Primary S Assimilation was Activated under AR

**Figure 4:**
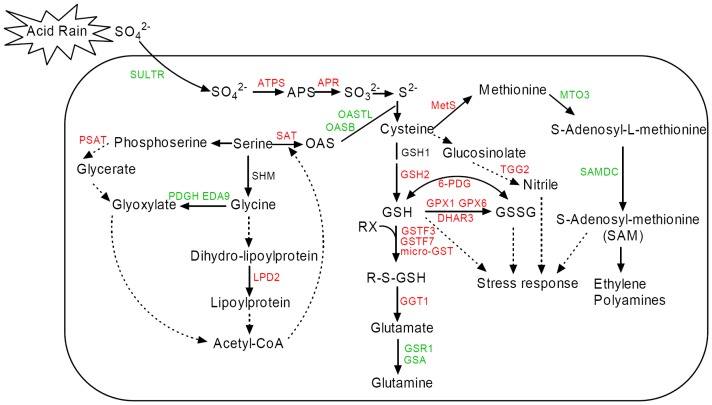
Schematic representation of a possible systematic response mechanism related to sulfur metabolism in *Arabidopsis thaliana* under simulated acid rain (AR) stress. The up- and down- regulated proteins or genes are labeled in red and green. The direct and indirect interaction of proteins or genes are indicated as solid or dotted line, respectively. Combined with our results, this figure was developed from the reviews of Hawkesford *et al*. [56] and Saito [11].

The combined proteomic and transcriptomic analysis on our experimental data sets provided a superior view of the complex physiology of *Arabidopsis* in response to AR compared to either proteomic or transcriptomic approach alone. As shown in Figure 4, the proteins/genes data were obtained from the proteomic and genomic microarray expreiments, which revealed a possible systematic AR-responsive mechacnism of S assimilation and related pathways in *Arabidopsis* under AR treatment.

Sulfate (SO_4_^2−^) is the most oxidized and thus a stable form of S presented in the soil. Uptake of S into roots from the soil is almost exclusively via sulfate uptake [8]. In our experiment, we found the expression of sulfate transporter gene (*SULTR 1;2*), which has an important function in S uptake, was reduced under AR treatment. A number of genes encoding the sulfate transporter have been reported in *Arabidopsis* [28]–[30]. They are classified into five subfamilies, named SULTR1 to 5, according to their deduced amino acid sequences. The members in SULTR1 are high-affinity transporters for sulfate. SULTR1;1 and SULTR1;2 of *Arabidopsis* are inducible by sulfate depletion, responsible for initial uptake of sulfate from outside of the plant cell [29]. The transporter is well known to show a strong repression in expression in the presence of an adequate S supply. Transport activity, mRNA pool size and protein expression all decrease under conditions of excess S supply [31], [32]. In our study, AR treatment increased soil sulfate, hence it is not surprising that sulfate transporter gene expression was down-regulated”.

For assimilation, sulfate must be activated by APS, in which sulfate is linked by an anhydride bond to a phosphate residue by consumption of ATP and concomitant release of pyrophosphate [33]. This reaction is catalyzed by APS and is the sole entry step for S metabolism. It is reported that APS mediates the reduction reaction of sulfate to sulfite by APS reductase (APR) in plants, which is subsequently reduced to sulfide by sulfite reductase [32]. Many studies have found that APR is another key enzyme in sulfate assimilation in plants [9], [34]. In our experiment, we found that the increase of APR mRNA accumulation contributes to the higher sulfate assimilation from outside into plant under AR treatment (Figure 4).

**Figure 5:**
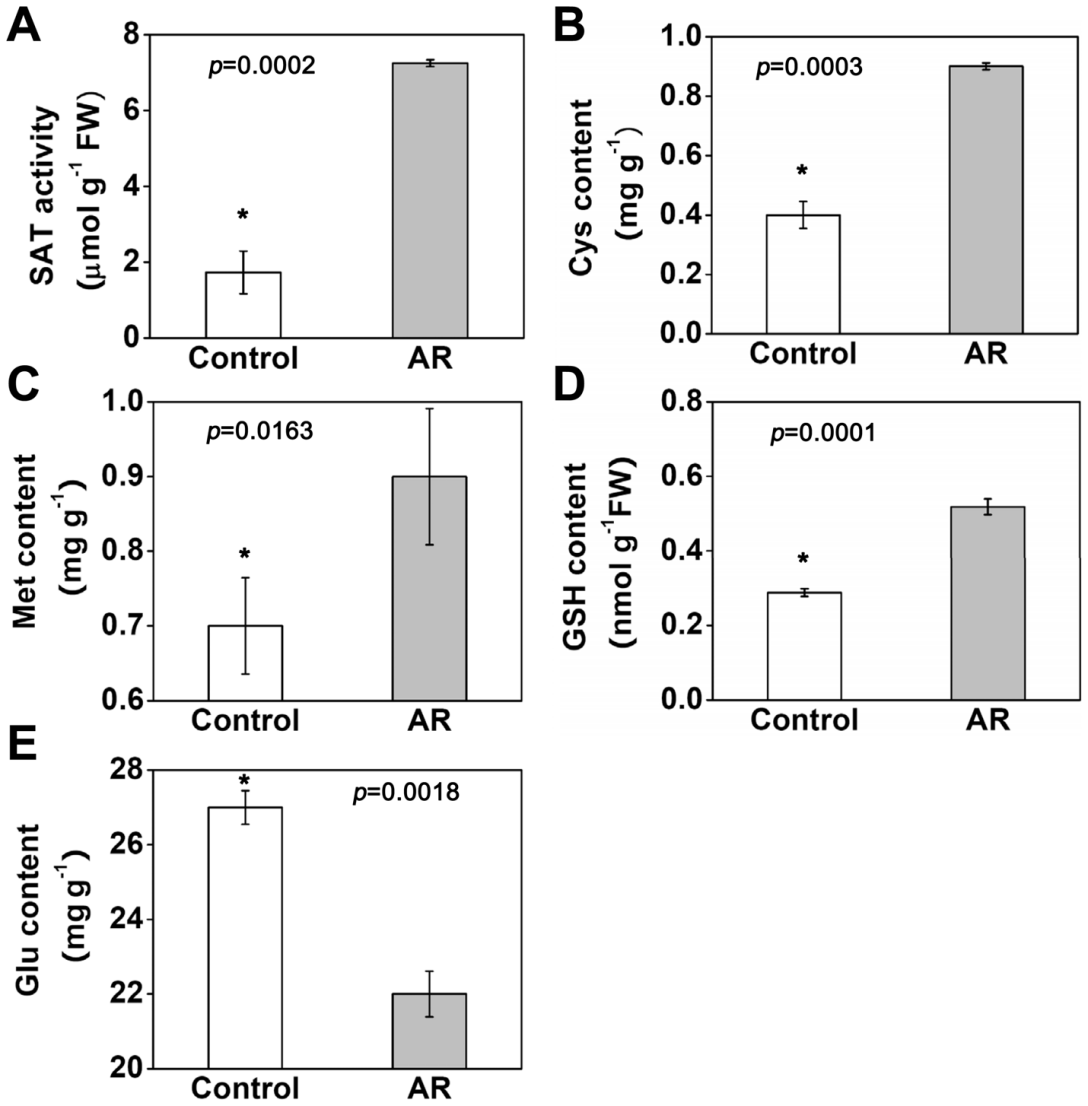
Effects of simulated acid rain (AR) on the reduced glutathione (GSH) content (A), Ser acetyltransferase (SAT) activity (B) and three S-containing amino acid contents (C–E) in *Arabidopsis thaliana*. The values were derived from the average of three replicate measurements. The asterisk indicates significance at *p*<0.05.

The final step in the assimilation of reduced sulfate is the incorporation of S into thiol-containing amino-acid, Cys [11]. Two enzymes, Ser acetyltransferase (SAT) and *O*-acetylserine (thiol) lyase (OASTL), are committed for this step. SAT catalyzes the formation of *O*-acetylserine (OAS) from Ser and acetyl-CoA. Many reports have found that SAT plays an important role in regulating Cys biosynthesis [10], [35], [36]. While the plants were exposed to AR, the expression in gene level of SAT was up-regulated, however, the OASTL and OASTL isoform oasB (OASB) were down-regulated. All of the expression changes lead to the increased assimilation of inorganic S into Cys. Consequently, the activity of SAT was also up-regulated under AR treatment due to high concentration of sulfate (Figure 5A). Meanwhile, higher level of Cys content was observed in our study (Figure 5B).

Cys is the pivotal sulfur-containing compound regarded as the terminal metabolite in S assimilation and the starting point for biosynthesis of Met, GSH, and a variety of other S-containing metabolites [32]. Therefore, the increased content of Cys eventually led to the increase in Met and GSH contents (Figure 5C and D), as well as the increase in expression of several Met and GSH biosynthesis related genes at transcriptional and protein levels (Figure 4) under AR.

### Downstream Genes and Proteins in S Metabolism Pathway Were Depressed by AR

Different from the primary S assimilation, where more Cys, Met and GSH synthesis process was induced under AR (Figure 4 and 5), the metabolism of some other amino acids and derivatives was depressed under AR. In our study, two Glu synthetase (GS) genes (glutamine synthase clone R2 gene, *GSR1*; glutamate-ammonia ligase gene, *GLD*) were down-regulated. GS is a key enzyme in this nitrogen assimilatory process, as it catalyzes the first step in the conversion of inorganic nitrogen (ammonium) into its organic form [37]. Consistently, we detected that the Glu content was greatly decreased under AR treatments (Figure 5E). These results suggested that nitrogen (N) assimilation was inhibited under AR treatments. On the other hand, glutamate-1-semialdehyde 2,1-aminomutase (GSA) is the first committed precursor of porphyrin synthesis in organelles and organisms that use the carbon (C) 5 pathway [38]. As we know, lots of important organic molecules such as chlorophyll are closely related with porphyrin, indicating the C fixation is influenced by AR through GSA mediated porphyrin synthesis pathway. Earlier studies have shown that AR could inhibit respiration and photosynthesis, and further inhibit plant growth [2], [39], which could be an indirect proof of our data. Generally, these results indicated that N and C metabolism were disordered under AR treatment.

In Met cycle, Met is converted to S−Adenosyl Methionine (SAM), which is a methyl donor for numerous reactions. SAM is also a substrate for ethylene, polyamine and phytosiderophore synthesis [40]. The expression of SAMS3, which is a key enzyme in SAM synthesis, was greatly inhibited by AR, suggesting that the Met cycle was influenced by AR treatment. Besides, S-adenosylmethionine decarboxylase gene (*SAMDC*) expression was also depressed under AR in our study (Figure 4). Recently, *Arabidopsis* mutant analysis has indicated that *SAMDC* is essential for plant polyamine biosynthesis pathway and play an important role in plant growth and development [41]. In plants, polyamines are not only important for both stress responses and developmental processes but also essential for plant survival [42]. The disorder of polyamines metabolism by AR would lead to more serious plant damage.

### GSH Plays a Crucial Role in Reactive Oxygen Species (ROS) Scavenging under AR

Cys availability has been shown to be the main factor limiting GSH production, both in normal plants and in those that overexpress genes for GSH biosynthesis [43]. Cys is incorporated into GSH that is one of the major redox controllers that plays significant roles in scavenging ROS through the GSH-ascorbate cycle [25] in which the dehydroascorbate reductase (DHAR) reduces dehydroascorbate to ascorbate, while oxidizing GSH to glutathione disulfide [27]. Many enzymes involved in this process were up-regulated in our study which led to the synthesis of more GSH in plant cells (Figure 4). Although some reports indicated that Cys and GSH are negative regulators of gene expression responding to S assimilation [44], [45], there were some other reports that the level of Cys increased in SO_2_ fumigated beech leaves [24]. In spruce trees, exposure to SO_2_ increased the accumulation of GSH and the activation of several scavenging enzymes [46]. An additional effect of SO_2_ fumigation was an increased level of sulfate suggesting that increased content of thiols in response to excessive S deposition is a common phenomenon.

As we know, ROS as a typical secondary stress triggered by AR, can cause severe damage to plants including growth and photosynthesis reduction, and premature senescence as well [47]–[49]. To prevent damage to membranes, chlorophylls and proteins, ROS have to be detoxified by scavenging systems that are consisting of low-molecular weight antioxidants and antioxidative enzymes in the apoplast and the symplast of plant cells [50]. Accumulating evidence further suggests that these adaptive responses of plants to increased ROS levels are mediated by changes in cellular GSH concentrations or the redox status of GSH pool [43].

Direct evidence showing ROS is a signaling component in plants is not yet available. However, several genes involved in S assimilation and synthesis of S-containing amino acids were induced by exposure to O_2_^−^ [48]. In this study, we identified a set of genes and proteins related to GSH metabolism pathways which were greatly up-regulated. For example, the expression of two genes *(GPX1*, *GPX6*) encoding glutathione peroxidases (GPX) was induced under AR in our study. As we know, GPX is the general name of the enzyme family with peroxidase activity whose main biological role is to protect the organism from oxidative damage. Evidence suggested that GPX activity also plays a role in stress-related signal transduction [51]. The plant glutathione transferases, formerly known as glutathione S-transferases (GSTs) are a large and diverse group of enzymes that catalyze the conjugation of electrophilic xenobiotic substrates with GSH [52]. Besides, GSTs are also important components of the cellular defense against oxidative stress [38]. We found GST genes or proteins were induced in both proteomic and transcriptomic experiments. Consistently, numerous studies have revealed that members of the GST super-family are expressed in response to microbial infection, cell division and environmental stresses [53], [54] as well as AR treatment in our pervious study [55].

Surely, acid rain not only disordered S metablism, but also effceted many other pathways. Lots of publications have demonstrated that AR causes a series of damages to plants, which includes destruction of the cell membrane, inhibition of respiration and photosynthesis, as well as disorders in metabolism of glucose, lipids and amino acids. Our transcriptomic and proteomic analysis also revealed that the expression of a set of genes and proteins related to primary metabolism, photosynthesis, metabolism of ROS, cellular transport, and signal transduction, were influenced by AR treatment. Due to the complexity of the emerging pattern, further work is required to delineate and confirm the precise effects of AR on metabolism and physiology.

## Conclusion

Using proteomic and transcriptomic methods, we studied the responses of S uptake and metabolic pathways in *Arabidopsis* seedlings exposed to simulated AR. By summarizing the information on the coordination between different metabolic changes, a network of mutual cross-influences in the AR-stress response could be assembled. Apparently, the entire network of S metabolism was coordinately regulated under AR stress. First of all, sulfate uptake and acquisition, that totally control the input of sulfate into S metabolic pathways, have been identified to be in positive correlation with AR. Furthermore, the activation of sulfate also increased as AR was imposed. Thirdly, the biosynthesis from sulfate to S-containing amino acid, for example, Cys and Met and other secondary metabolites were up-regulated under AR stress. Finally, we depicted the coordinating network of S metabolism including S uptake, activation, S-containing amino acid biosynthesis and other S-containing metabolites synthesis under AR stress. This study can help us to understand the mechanisms by which plants adapt to AR environment by alteration of the S metabolism.

## Supporting Information

Figure S1 Injury phenotype of Arabidopsis leaves under simualted acid rain treatment.
(PDF)

Table S1 Primer pairs used in qRT-PCR analysis for 12 sulfur metabolism related genes. Actin 2 was used as a standard to normalize the content of cDNA.
(DOC)

Table S2 The details of identified acid rain stress-responsive proteins in Arabidopsis.
(DOC)
