# Supplementary material for: A Combined Proteomic and Transcriptomic Analysis on Sulfur Metabolism Pathways of Arabidopsis thaliana under Simulated Acid Rain
Source: PLoS One. 2014 Mar 3;9(3):e90120. doi: 10.1371/journal.pone.0090120 (PMC3940841; doi:10.1371/journal.pone.0090120)
Supplement: Figure S1 — Injury phenotype of Arabidopsis leaves under simualted acid rain treatment. (PDF) [file pone.0090120.s001.pdf]

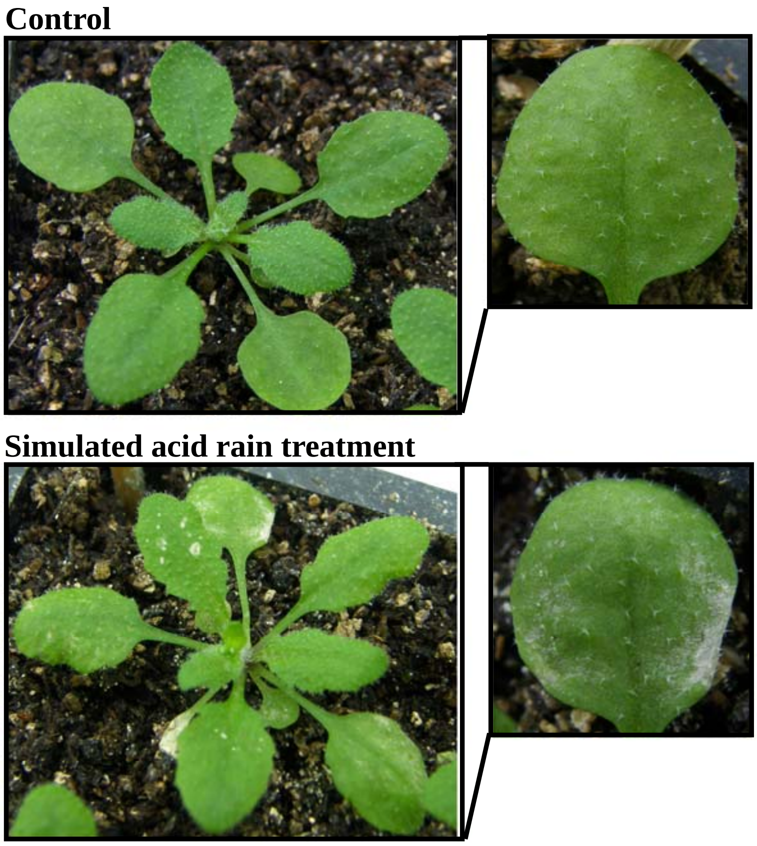

Figure S1: Injury phenotype of Arabidopsis leaves under simualted acid rain treatment
