# Supplementary material for: A Combined Proteomic and Transcriptomic Analysis on Sulfur Metabolism Pathways of Arabidopsis thaliana under Simulated Acid Rain
Source: PLoS One. 2014 Mar 3;9(3):e90120. doi: 10.1371/journal.pone.0090120 (PMC3940841; doi:10.1371/journal.pone.0090120)
Supplement: Table S1 — Primer pairs used in qRT-PCR analysis for 12 sulfur metabolism related genes. Actin 2 was used as a standard to normalize the content of cDNA. (DOC) [file pone.0090120.s002.doc]

| **ID** | **Name** | **Degenerated Oligo Nucleotide Primer Sequence** | **Orienation** |
| --- | --- | --- | --- |
| 1 | *Sulfate transporter1;2 (SULTR1;2)* | 5' TCACCCACTCGGCATAAAGTTGGAATC 3' | Sense |
|  |  | 5'GAAGACGGATTGGAGACCGAGCATAAACTG3' | Antisense |
| 2 | *APS reductase (APR2)* | 5'AGGGTGATGAAGAATGAAAGAGAATT3' | Sense |
|  |  | 5'AAAGTAGACCAAAAAAGATACATAGAGG3' | Antisense |
| 3 | *ATP sulfurylase (APS1)* | 5'CAAAGAACAACGAAAACCCGCCAGAC3' | Sense |
|  |  | 5'TGCTACAAGGCTTCACAACACAAACG3' | Antisense |
| 4 | *O-acetylserine(thiol)lyase (OASA1)* | 5'CCGTGCTCTAGCGTCAAAGACAGGA3' | Sense |
|  |  | 5'CTTGTTGGCTCAATCAGCACACTCTCT3' | Antisense |
| 5 | *Cystein synthase (OASB)* | 5'TTACCTCTCGACCCAGCTTTTCCAG3' | Sense |
|  |  | 5'GGTTCCTTGTTGGTCACGAAATTACAGA3' | Antisense |
| 6 | *Glutathione systhetase (GSH2)* | 5'CCTGATGCAGAGGATATTCCCAAAAGT3' | Sense |
|  |  | 5'AGCACCATAGACACCGAGTTCTGATAT3' | Antisense |
| 7 | *Glutathione S-transferase (ATGSTF3)* | 5'TTTCACTTTGACCGATCTTCACCACAT3' | Sense |
|  |  | 5'ATCTCAGCCACCCACTCGTTGACACG3' | Antisense |
| 8 | *Glutathione peroxidase (ATGPX6)* | 5'TGCGAAACCACTTTTCAATTCTCATC3' | Sense |
|  |  | 5'AGAGATTTGGGTTCGGAAGAAGCAG3' | Antisense |
| 9 | *S-adenosylmethionine synthetase(MTO3)* | 5'AGACTGCTGCCTATGGTCACTTTGGAA3' | Sense |
|  |  | 5'TTGGACCTTGTTAGACTTGAGTGGCTTG3' | Antisense |
| 10 | *S-adenosylmethionine decarboxylase(SAMDC2)* | 5'AGCGAGGACGAGAAGGAATAGGGAATC3' | Sense |
|  |  | 5'CAGACACAAACACTGGTTTAAGGGAAA3' | Antisense |
| 11 | *Myrosinase (TGG2)* | 5'TGTCTCTTCTTTCTACTTGCTCCATA3' | Sense |
|  |  | 5'CGAGTTTAGTTCAATTTTGGTTTCAT3' | Antisense |
| 12 | *Cytosolic thioredoxin (ATTRX5)* | 5'CACCTTGCCGTTTCATTGCACCAGT3' | Sense |
|  |  | 5'CATCTTTCGCAGCACCGACAACACG3' | Antisense |
|  | *Actin 2* | 5' AACTCTCTGGGTTTTTACTTACGTCTGCG3' | Sense |
|  |  | 5'AGGGAACAAAAGGAATAAAGAGGCATCAA3' | Antisense |

**Supplementary Table S1**. Primer pairs used in qRT-PCR analysis for 12 sulfur metabolism related genes. *Actin 2* was used as a standard to normalize the content of cDNA.
