# Supplementary material for: A Combined Proteomic and Transcriptomic Analysis on Sulfur Metabolism Pathways of Arabidopsis thaliana under Simulated Acid Rain
Source: PLoS One. 2014 Mar 3;9(3):e90120. doi: 10.1371/journal.pone.0090120 (PMC3940841; doi:10.1371/journal.pone.0090120)
Supplement: Table S2 — The details of identified acid rain stress-responsive proteins in Arabidopsis. (DOC) [file pone.0090120.s003.doc]

| **Spot no**  **Supplemental Table S2** The details of identified acid rain stress-responsive proteins in Arabidopsis.. | **Theo. M_r_ (kDa)/p*I*** | **Expt. M_r_ (kDa)/p*I*** | **Coverage** | **Score** | **ID** | **SP/TP** | **p-value** | **Change**  **(Log2)** | **Identity** | **MS peptide sequencee** |
| --- | --- | --- | --- | --- | --- | --- | --- | --- | --- | --- |
| P1 | 34/5.9 | 42/7.0 | 32% | 88 | AT4G14880 | 5/13 | 1.10E-03 | -1.85 | O-acetylserine (thiol) lyase (OASA1) | VAAKLEMMEPCSSVK  DRIGFSMISDAEK  LIITMPASMSTER  IDGFVSGIGTGGTITGAGK  YLSTVLFDATR |
| P2 | 42/8.2 | 42/7.0 | 21% | 89 | AT2G43750 | 6/15 | 9.80E-06 | -2.01 | (OAS-TL) isoform oasB (OASB) | MAATSSSAFLLNPLTSRHR  PEAGVEGLNIADNAAQLIGK  LEIMEPCCSVKDR  VESTSGNTGIGLAFIAASK  IDILVAGIGTGGTITGVGR  PENAGKLIAVVFPSFGER |
| P3 | 51/6.8 | 51/6.3 | 23% | 91 | AT3G22890 | 7/13 | 4.80E-16 | 1.24 | ATP sulfurylase (APS) | TPFLSQPLTK  SSPNSDLPFAAVSFPSKSLR  IAPDGGKLVELIVEEPK  VELTAIDLQWMHVLSEGWASPLGGFMR  LDRFRLSPAELR  ADDVPLDWR  DLYDADHGK |
| P4 | 53/5.5 | 54/5.6 | 19% | 71 | AT5G41670 | 6/16 | 1.60E-04 | 1.81 | 6-phosphogluconate dehydrogenase family protein (6-PDG) | IGLAGLAVMGQNLALNIADK  TTSKVDETLDR  IVEAEK  GLLYLGMGVSGGEEGAR  NGPSLMPGGSFTAYNNVK  EDIGSASR |
| P5 | 29/7.9 | 28/7.0 | 41% | 68 | AT5G16710 | 11/31 | 6.00E-13 | 2.13 | glutathione dependent dehydroascorbate reductase (DHAR3) | FQPSTTAGVLSASVSR  AGFIK  CGSTKPGR  ASITTPNK  LGDCPFCQK  MVDLSNKPEWFLK  VPVVKFDEK  WVPDSDVITQALEEK  LYHMKIALGHYK  NWSVPDSLPFVK  SYMENVFSR |
| P6 | 24/6.6 | 24/6.1 | 50% | 67 | AT1G02920 | 8/29 | 1.20E-09 | 1.52 | glutathione S-transferase (GST3) | VFGHPASTATR  KEPFIFR  EPFIFR  VPAFEDGDFKLFESR  AITQYIAHFYSDKGNQLVSLGSK  DIAGIAMGIEIESHEFDPVGSK  VLDVYEHR  LFDERPHVSAWVADITSRPSAK |
| P7 | 43/5.6 | 43/5.5 | 46% | 95 | AT3G17390 | 15/32 | 7.60E-09 | -1.23 | S-adenosylmethionine synthetase (MTO3) | MESFLFTSESVNEGHPDK  LCDQISDAILDACLEQDPESK  VACETCTK  ANVDYEQIVR  TCREIGFVSADVGLDADNCK  LGAKLTEVR  NGTCPWLR  TQVTIEYINESGAMVPVR  VHTVLISTQHDETVTNDEIAADLK  PVIPEKYLDEK  FVIGGPHGDAGLTGR  IIIDTYGGWGAHGGGAFSGK  DPTKVDR  SGAYIVR  SIVASGLAR |
| P8 | 84/6.5 | 85/6.1 | 31% | 139 | AT5G17920 | 17/24 | 1.50E-04 | 1.13 | Methionine synthase (MetS) | MASHIVGYPR  RELKFALESFWDGK  VSADLRSSIWK  FAHYDQVLDTTAMLGAVPPR  YGYTGGEIGLDVYFSMAR  NASVPAMEMTK  WFDTNYHYIVPELGPEVNFSYASHK  ALGVDTVPVLVGPVSYLLLSK  SFELLSLLPKILPIYK  EVITELK  STLSGLNVLVETYFADIPAEK  TLTSLKGVTAFGFDLVR  VSEEDYVK  VVDLQEELDIDVLVHGEPER GEQLSGFAFTANGWVQSYGSR  PPVIYGDVSR  YGAGIGPGVYDIHSPR |
| P9 | 53/6.9 | 49/6.6 | 24% | 87 | AT1G23310 | 12/36 | 1.20E-03 | 1.04 | glutamate-glyoxylate aminotransferase (GGT1) | ALDYDTLNENVK  GELYLRASELQK  HYLSLTSGGLGAYSDSR  EVAEFIQR  DGYPSDPELIFLTDGASK  GVMQILNCVIR  SQGITVR  LVLLGDEVYQQNIYQDER  EVQLVSFHTVSK  GYWGECGQR PGDISYDQFAR  LMTDGFNSCK |
| P10 | 39/4.9 | 39/5.1 | 28% | 95 | AT1G66200 | 10/17 | 1.50E-02 | -1.28 | glutamine synthase clone R2 (GSR2) | MSLLADLVNLDISDNSEK  IIAEYIWVGGSGMDMR  TLPGPVTDPSK  SFGRDVDAHYK  YILERITEIAGVVVSFDPK  PIPGDWNGAGAHTNYSTK  EEGGYEIIK  LTGHHETADINTFLWGVANR  VGRDTEK  EGKGYFEDR |
| P11 | 39/5.9 | 39/5.7 | 47% | 92 | AT3G17820 | 14/22 | 6.10E-07 | -1.59 | glutamate-ammonia ligase (GLD) | MSLLSDLVNLNLTDATGK  IIAEYIWIGGSGMDIR  TLPGPVTDPSK  WNYDGSSTGQAAGEDSEVILYPQAIFK  GNNILVMCDAYTPAGDPIPTNKR  IFSHPDVAK  EEPWYGIEQEYTLMQK  PGPQGPYYCGVGADK  AIGRDIVDAHYK  QVGPVEGISSGDQVWVAR  ITEISGVIVSFDPK  PVPGDWNGAGAHCNYSTK  NDGGLEVIK  EHIAAYGEGNER |
| P12 | 50/6.9 | 51/6.4 | 27% | 99 | AT5G63570 | 11/27 | 1.50E-13 | -1.98 | glutamate-1-semialdehyde 2,1-aminomutase (GSA) | MSATLTGSGTALGFSCSSK  VSSSPASNR  SEEAFNAAK  NLMPGGVNSPVR  LAEMVISAVPSIEMVR  FVNSGTEACMGVLR  AFTNKEK  DNGVLLIFDEVMTGFR  IIGGGLPVGAYGGR  QAGTYEYLDK  TGHPMCGGYISGMFGFFFAEGPVYNFADSK |
| P13 | 54/7.0 | 54/6.6 | 32% | 86 | AT3G17240 | 10/23 | 5.60E-03 | 1.56 | lipoamide dehydrogenase (LPD) | AYFLTRNISNSPTDAFR  AAQLGLKTTCIEKR  GALGGTCLNVGCIPSK  VSSVEVDLPAMLAQK  DTAVKNLTR  FLSPSEVSVDTIDGENVVVK  VVGVDSSGDGVK  ACVEFIAGK  EGVSYNVGKFPFMANSR  NYDASSEDIAR |
| P14 | 63/6.5 | 64/6.3 | 23% | 106 | AT4G34200 | 15/27 | 6.50E-02 | -1.74 | phosphoglycerate dehydrogenase (EDA9) | MSATAAASSSIAVATNSLR  SPLPSAISVAFPSR  PTILVAEK  LGDAGIK  ISLCDALIVR  SGTKVGR  EVFESSHGR  TIAAAEHGIALMAAMAR  NVAQADASVKAGEWKR  YVGVSLVGK  GGVIDEDAVR  DSKLVQHER  PYVVLAEKLGR  LAVQLVAGGSGVK  ITYASAR |
| P15 | 20/9.6 | 21/6.9 | 34% | 90 | AT4G03520 | 5/9 | 2.40E-09 | 2.01 | thiol-disulfide exchange intermediate (TRX5) | ATGPVVVDFWAPWCGPCK  MIDPLVNDLAQHYTGK  LNTDESPNTPGQYGVR  SIPTIMIFVGGEK  SIPTIMIFVGGEK |
| P16 | 63/7.5 | 62/7.1 | 33% | 180 | AT5g25980 | 14/21 | 2.10E-04 | 1.86 | thioglucoside glucohydrolase (TGG2) | FNKQDFESDFIFGVASSAYQIEGGR QDFESDFIFGVASSAYQIEGGR GLNVWDGFTHR GLNVWDGFTHRYPEK FSFAWSR GINEDGINYYSGLIDGLIAR TIIDDFKDYADLCFER VKHWITINQLFTVPTR HWITINQLFTVPTR GYALGTDAPGR CYGGDSSTEPYNVAHNQLLAHATVVDLYR WFLPYDDTLESK GKYPYIMR IDYLCSHLCFLR |
